# Clinical and psychopathological follow-up of patients with anorexia nervosa with or without NSSI comorbidity admitted to an inpatient service

**DOI:** 10.1007/s40519-026-01850-8

**Published:** 2026-04-09

**Authors:** Federico Amianto, Beatrice Ferrero, Chiara Davico, Daniele Marcotulli, Andrea Martinuzzi

**Affiliations:** 1 Neurosciences Department, University of Torino, Via Cherasco 15, 10126 Turin, Italy; 2 Department of Public Health and Pediatric Sciences, University of Turin, 10126 Turin, Italy

**Keywords:** Anorexia nervosa, NSSI, Adolescence, Personality, Psychopathology, Outcome

## Abstract

**Purpose:**

Approximately 27% of patients with an eating disorder (ED) present with comorbid non-suicidal self-injurious behaviors (NSSI). EDs comorbid with NSSI display worse eating psychopathology and overall psychological distress. This follow-up study aims to compare the clinical and psychopathological characteristics at the time of hospital admission and the long-term outcome of patients with and without comorbid NSSI.

**Methods:**

73 adolescents affected with an ED were included in the study and divided into two groups depending on the comorbidity with NSSI. Clinical and anamnestic data, personality traits, social, emotional and behavioral functioning, and eating psychopathology were collected. A comparison was made between the NSSI group and the non-NSSI group on clinical and psychopathological characteristics. After a 5-year follow-up, participants were interviewed with a structured interview to investigate the history of AN and NSSI, and they completed questionnaires for the self-assessment.

**Results:**

Specific personality traits like low Cooperativeness and low levels of Reward Dependence, greater impairment of behavioral and emotional functioning, and worse eating psychopathology characterized AN participants with NSSI. The 5-year outcome was less favorable in participants with a history of NSSI with a remission rate 77.7% in participants with AN alone and of 21% in participants with NSSI.

**Conclusions:**

The differences between subgroups highlight the need to investigate NSSI at the initial clinical evaluation of adolescents who come to observation for an ED. Clinical and psychopathological characteristics of AN with comorbid NSSI require more intensive care and tailored therapeutic approaches to improve the long-term outcome, promoting recovery and improving quality of life.

*Level of Evidence* III: Evidence obtained from well-designed cohort or case–control analytic studies

## Introduction

Anorexia nervosa (AN) and non-suicidal self-injury (NSSI) are both conditions involving self-damaging behaviors, although they differ in underlying mechanisms, including biological, psychological, and starvation-related processes in AN. They display typical onset during adolescence, higher prevalence among females, and a strong association with altered personality traits [1]. Approximately 27% of patients with an eating disorder present with comorbid NSSI, with a stronger association observed in the Binge–Purging subtype of Anorexia Nervosa compared to the Restrictive subtype [2].

The high comorbidity rate is attributed to shared individual and social risk factors, similar underlying functions, and common predisposing psychological characteristics, such as emotion regulation difficulties, specific personality traits, and identity formation issues [3].

In most cases, the onset of NSSI follows the onset of the eating disorder, but the age of onset for the eating disorder is earlier in those with NSSI compared to those without NSSI [4].

Adolescents presenting both disorders in comorbidity show similar or better physical health compared to those with the eating disorder only; however, they exhibit more severe eating psychopathology and greater psychological distress [5].

Few studies explore the interaction between NSSI and the long-term outcomes of eating disorders. A higher prevalence of NSSI appears to be associated with a longer duration of the eating disorder and of the time needed for inpatient treatment [6].

In line with the literature [1] patients with AN and NSSI present a more severe psychopathology, more compromised overall functioning, and a more unstable structure of the self, reflected in greater immaturity of character, i.e., lower Self-Directedness (SD) and Cooperativeness (C) compared to AN-only patients.

The aim of the present study was to follow up two groups of AN adolescents, one with and the other without comorbid NSSI, 5 years after an inpatient treatment to explore the differences in their clinical evolution.

Given the greater overall psychopathological impairment in AN patients with NSSI [1] and the literature evidence of a longer duration of inpatient treatment in these patients [6], it is likely that the long-term outcome for AN patients with NSSI is worse compared to AN without a history of self-harming behaviors [7].

On the other hand, considering the high frequency of spontaneous remission of the NSSI after 5 years from their beginning, we expect that most of these patients (80%) will no longer engage in self-injurious behaviors [8].

## Materials and methods

### Sample description

For the present study, all the adolescents affected with AN consecutively hospitalized in the Child and Adolescent Neuropsychiatry Inpatient Unit and in the Pediatric Wards of the Regina Margherita Hospital in Turin between January 1, 2018 and December 31, 2019 were considered.

The inclusion criteria were as follows: a diagnosis of Anorexia Nervosa (AN) according to the DSM-5-TR (2023) diagnostic criteria, female gender, age between 12 and 17 years at the time of diagnosis. Underweight status was determined using a BMI fixed threshold of 18.5. Body Mass Index (BMI) was recorded at admission. Although age-adjusted BMI measures are recommended in adolescents, retrospective data availability limited their use.

The exclusion criteria were as follows: a diagnosis of Atypical AN or ARFID, comorbid ASD, intellectual disability, acute psychosis or bipolar disorders. Exclusion criteria were applied to reduce clinical heterogeneity and avoid confounding effects on psychometric assessment of severe neurodevelopmental and psychiatric conditions.

The sample recruited for the follow-up was identified through a database search at the Regina Margherita Children’s Hospital in Turin. The parents of the adolescents were contacted with a telephone call by a member of the research staff who explained the aims, procedures and ethical implications of the research and asked to sign the written informed consent prior to the clinical interview with the children.

During hospitalization, all participants received care from a multidisciplinary team comprising child neuropsychiatrists, psychologists, psychotherapists, and nutritionists. The treatment approach was integrated, including pharmacological, psychotherapeutic, and dietary interventions tailored to individual needs.

### Study phases

This study is a two-phase follow-up of a clinically homogeneous sample based on diagnosis. The recruited sample included 73 inpatients who met the inclusion criteria. Out of the 73 participants contacted during follow-up, 12 were unreachable, 15 declined to participate, 46 participated in the interview, and 35 of them returned adequately completed questionnaires. Due to the eminent clinical aims of the research, all interviewed participants were included in the study.

At intake (T0) in the study anamnestic and clinical data were extracted from clinical records, including: age at hospitalization, age of AN onset, AN subtype, body mass index (BMI), comorbidity with other mental disorders, presence of NSSI. These last were categorized as R-NSSI (Repetitive-NSSI) if at least five self-injurious episodes occurred within a year, or O-NSSI (Occasional-NSSI) if less frequent. Frequency and methods of NSSI were also collected. The results of the psychodiagnostic questionnaires TCI, EDI-II, and YSR were also retrieved.

The follow-up (T1) was conducted 4–5 years after inpatient discharge between January 1, 2023, and December 31, 2024. Participants were contacted by telephone, and those who consented participated in a structured interview designed to assess their current health status, including weight, BMI, desired weight, presence/absence of dysfunctional eating thoughts or behaviors, and presence/absence of NSSI. The psychodiagnostic questionnaires were administered for comparison with the T0 data.

AN status was classified as ‘active illness’ when all DSM-5 criteria for AN were still met; ‘partial remission’ was classified when BMI was within the normal range, but dysfunctional thoughts or behaviors persist; ‘full remission’ was classified when no DSM diagnostic criteria were met, and the patient was considered fully recovered.

Other variables assessed included the presence, frequency, and methods of NSSI in participants who still engaged in these behaviors at T1, the total duration of NSSI in participants who had ceased NSSI by T1, and the presence of Axis I Disorders.

### Assessment tools

Psychodiagnostic questionnaires administered at T0 and T1 included the Temperament and Character Inventory (TCI) for personality assessment, the Youth Self-Report (YSR) for general functioning and psychopathology assessment and the Eating Disorder Inventory-II (EDI-II) for eating psychopathology assessment.

Temperament and character inventory (TCI): The TCI is a self-administered questionnaire with 240 true/false items that assess personality dimensions [9]. According to Cloninger’s psycho-biological model, personality is divided into four temperamental dimensions (Novelty Seeking, Harm Avoidance, Reward Dependence, and Persistence), which are genetically determined and relatively stable over time, and three character dimensions (self-directedness, cooperativeness, and self-transcendence), which evolve with age and maturation processes and are influenced by environmental and sociocultural factors.

Youth self-report (YSR): The YSR is a self-report questionnaire for adolescents aged 11–18 that assesses social competencies, academic performance, activities, general functioning, emotional reactions and behavioral problems [10]. It provides two profiles: one for competencies and one for behavioral/emotional problems. Scores fall into “normal,” “borderline,” or “clinical” ranges based on specific syndromic scales:

- *Internalizing problems scale*: includes withdrawal, somatic complaints, and anxiety/depression.
- *Externalizing problems scale*: includes rule-breaking and aggressive behavior.
- *Other problem scales*: social problems, thought problems, and attention problems.

Eating disorder inventory-II (EDI-II). The EDI-II is a self-report questionnaire with 91 items assessing traits critical for understanding eating disorders [11]. It measures 11 scales: Drive for Thinness (DT), Bulimia (BU), Body Dissatisfaction (BD), Ineffectiveness (IN), Perfectionism (PF), Interpersonal Distrust (ID), Interoceptive Awareness (IA), Maturity Fears (MF), Asceticism (ASC), Impulse Regulation (IR) and Social Insecurity (SI).

### Ethical issues

All participants and their parents gave written informed consent for participation in the study. The study was approved by the local ethical committee (Comitato Etico Territoriale di Torino—CET) with the protocol number: 0009691.

### Statistical analysis

The statistical analysis was performed using SPSS 29.0 (Statistical Package for Social Sciences 29.0).

This prospective observational study compares the clinical and psychopathological characteristics at the time of the hospital admission (T0) and at a 4–5 year follow-up (T1) between two groups of participants: participants with AN alone and participants with AN comorbid with NSSI at intake.

The chi-square test was used to compare the two groups for nominal variables: diagnostic subtype of Anorexia Nervosa (Restrictive, Binge–Purging, and Atypical) and the compensatory physical hyperactivity.

The Student’s *t* test was used to compare the participants with NSSI alone and an comorbid with NSSI at intake groups for ordinal or continuous variables at T0: AN age of onset, BMI, length of hospitalization, TCI, YSR, and EDI-II scores.

AN adolescents who consented to participate in the follow-up, and those who did not were also compared to test the representativeness of the sample participating in the study.

AN comorbid with NSSI at intake participants were compared to those reporting NSSI episodes during the period between hospitalization (T0) and the follow-up (T1). The groups were compared using the chi-square test for weekly frequency of NSSI and the total number of methods used. Student’s *t* test was applied to compare AN age of onset, BMI, length of hospitalization, TCI, YSR, and EDI-II scores.

Follow-up participants were divided into two groups: AN and AN with NSSI. The chi-square test was used to compare the AN rate of remission between the two groups. Participants in the AN with NSSI group were compared for AN remission between those with NSSI still present at T1 and those who no longer engaged in NSSI at T1.

At follow-up, due to the non-normal distribution of the variables consequent to the limited number of participants who filled in the psychometric measures both at T0 and T1, the Mann–Whitney *U* test was applied to compare continuous variables (TCI, YSR, and EDI-II). The T0–T1 differences (deltas) in BMI, TCI, YSR, and EDI-II scores were calculated and compared between groups using the Student’s *t* test.

Information on NSSI frequency and methods was collected to provide a descriptive characterization of self-injurious behaviors. Given the limited sample size, these variables were not included in inferential analyses. Future analysis on larger samples may permit this exploration.

Information on suicide attempts was not systematically available in clinical records and at follow-up and was, therefore, not included in the present analyses.

## Results

### Sample description at T0

#### NSSI prevalence and follow-up participation

**Fig. 1:**
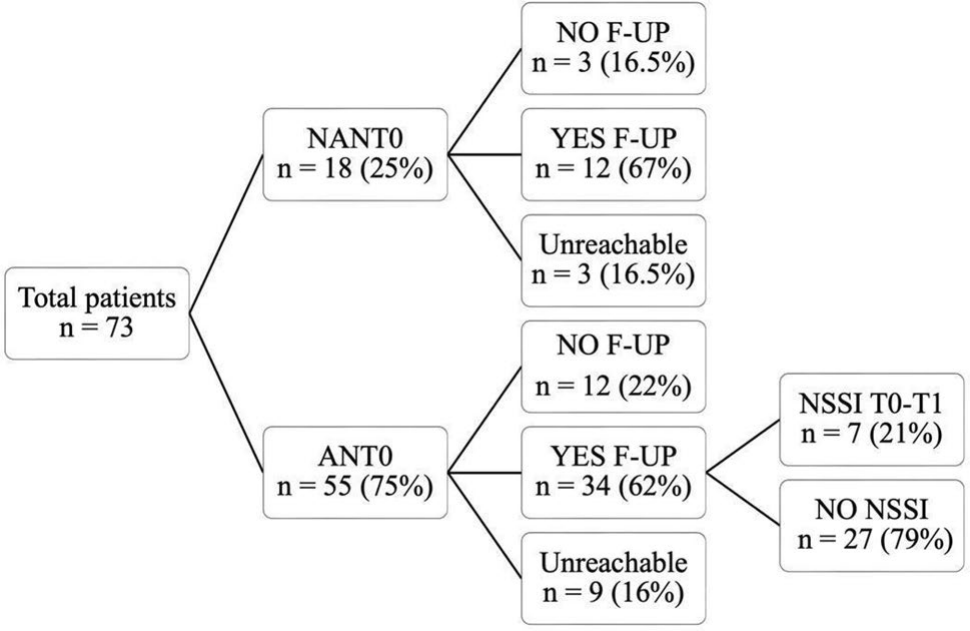
Flow-chart NSSI prevalence and follow-up participation

Figure 1 describes the flow chart of the study. Among the 73 participants who met the recruitment criteria, 18 (25%) were diagnosed with anorexia nervosa (AN) comorbid with non-suicidal self-injury (NSSI) at the time of hospitalization at OIRM (AN comorbid with NSSI at intake). In the AN comorbid with NSSI at intake group, 12 participants (67%) consented to participate in the follow-up, 3 (16.5%) did not consent, and 3 (16.5%) were unreachable. Among the participants with AN alone group, 34 (62%) consented to participate, 12 (22%) did not consent, and 9 (16%) were unreachable.

The mean age of onset for Anorexia Nervosa was 14 years, with no significant difference between the ANT0 and AN comorbid with NSSI at intake groups; similarly, the mean age of onset for NSSI was also 14 years.

Seven participants (21%) engaged in NSSI between hospitalization and follow-up (T0–T1). At T1 25 (34%) participants had experienced at least one episode of NSSI.

#### NSSI type, frequency, and methods

Tables 1 and 2 describe the distribution of the NSSI frequency and methods among participants. Repetitive-NSSI (R-NSSI) is observed in 64% of cases.

**Table 1:** NSSI weekly frequency

	*N*	Rate (%)
Less than once	9	41
1–2 times	6	27
More than twice	7	32

**Table 2:** NSSI methods

	*N*	Rate (%)
Cutting	17	77
Scratching	12	64
Punching of slapping	7	32
Skin carving	5	23
Burning	3	14
Biting	3	14
Preventing wound healing	3	14
Head banging	1	4

Out of 22 participants who reported the weekly frequency of NSSI, 43.8% of participants reported engaging in NSSI at least twice a week, with the most used methods being cutting (77%), scratching (64%) and punching or slapping (32%). Out of 22 participants, 16 (72.7%) engaged in two or more self-injurious methods.

#### Comorbid disorders

A total of 29 participants (39.7%) had a concurrent psychiatric disorder at the time of hospitalization. A major depressive episode was diagnosed in 19 participants (65.5%), followed by obsessive–compulsive disorder (OCD), diagnosed in 7 participants (24%). Other concurrent disorders were social anxiety, diagnosed in 2 participants, and borderline personality disorder, diagnosed in 1 patient. 

### Sample description at T1

#### Remission rates of AN

Table 3 displays the rate of remission from AN in the AN with NSSI and AN groups. In the AN with NSSI group (*n* = 19), 7 participants (37%) still met all diagnostic criteria for AN, 8 participants (42%) were in partial remission, 4 participants (21%) were in full remission. In the AN group (*N* = 27), 2 participants (7%) still met diagnostic criteria for AN, 4 participants (15%) were in partial remission, 21 participants (78%) were in full remission.

**Table 3:** AN remission rate

	AN (*n* = 27)	AN with NSSI (*n* = 19)
Active illness	2 (7%)	7 (37%)
Partial remission	4 (21%)	8 (42%)
Full remission	21 (78%)	4 (21%)

*χ*^2^ Test score = 14.7; df = 2; *p* < 0.001

**Fig. 2:**
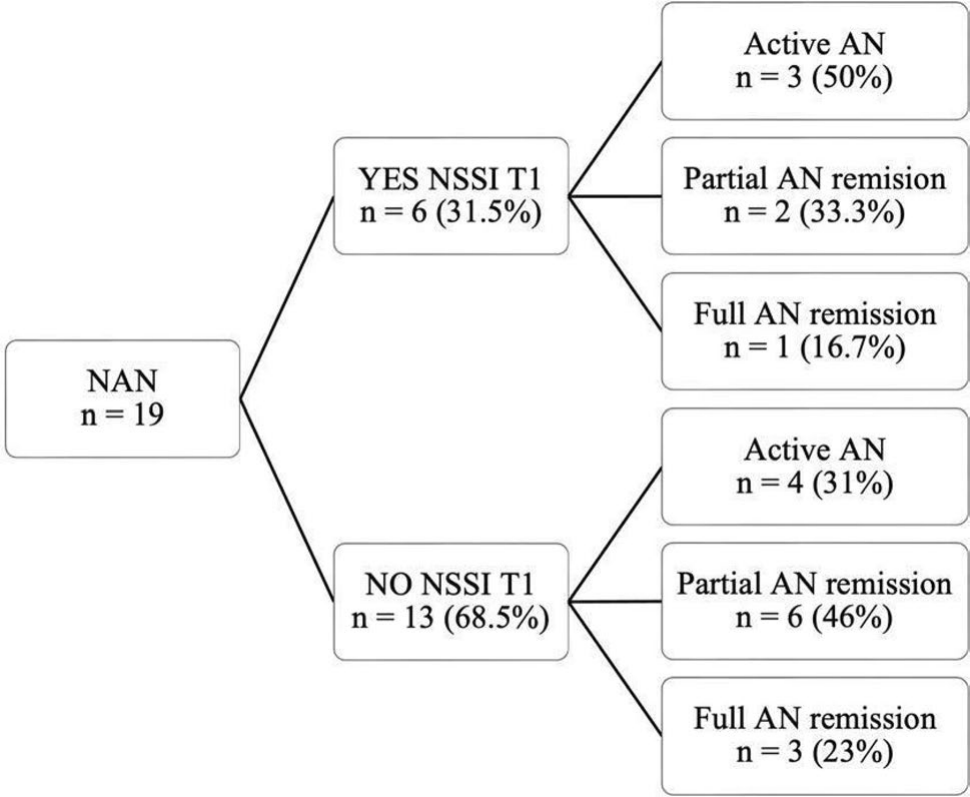
AN remission categorized by presence or absence of NSSI at T1. The figure displays the flow chart of AN remission categorized by presence or absence of NSSI at T1. NAN = AN with NSSI

Figure 2 displays the rates of AN remission categorized by presence or absence of NSSI at T1. In the AN with NSSI group, 6 participants (31.5%) continued to engage in NSSI, and among them, 3 (50%) had stable AN; 13 participants (68.5%) no longer engaged in NSSI, among these, 4 participants (31%) had stable AN. The average duration of NSSI ranged between 4 and 5 years, with a minimum duration of 6 months observed in one patient and a maximum duration of 8 years.

#### Comorbid disorders

Among participants in the AN with NSSI group 11 participants (58%) had a comorbid disorder: 6 participants (54.5%) were diagnosed with Borderline Personality Disorder (BPD), 1 patient with bipolar disorder, 2 participants with Major Depression, 1 patient with Generalized Anxiety Disorder and 1 patient with OCD. In the AN group 6 participants (22%) had a comorbid disorder: Major Depression (1 patient), Generalized Anxiety Disorder (1 patient), Panic Disorder (3 participants) and OCD (1 patient).

### Comparison between participants and nonparticipants

No significant differences emerged between the participant and nonparticipant groups regarding AN subtype, NSSI, age of AN onset, hospitalization length and BMI. Therefore, the sample of participants who consented to participate in the follow-up can be considered representative of the overall study sample.

### Participants with AN alone vs AN comorbid with NSSI at intake comparison

No clinical characteristics (age of AN onset, hospitalization length and BMI) significantly distinguished the participants with AN alone and AN comorbid with NSSI at intake groups. 

Table 4 displays the comparison of the psychodiagnostic features. The AN comorbid with NSSI at intake group scored lower in Reward Dependence (RD) (*p* < 0.001), Helpfulness (C3) (*p* < 0.001) and Cooperativeness (C) (*p* < 0.001) at TCI.

**Table 4:** Student’s *t* test comparison of psychodiagnostics features between participants with AN alone and AN comorbid with NSSI at intake

TCI	ANT0 (*n* = 55) mean ± sd	NANT0 (*n* = 18) mean ± sd	*t*	*p*
Reward dependence (RD)	14.71 ± 3.49	9.29 ± 3.14	3.769	< 0.001
Helpfulness (C3)	6.46 ± 0.87	3.67 ± 1.03	6.118	< 0.001
Cooperativeness (C)	33.74 ± 5.2	24.00 ± 9.88	3.728	< 0.001
YSR				
Anxiety/depression	10.26 ± 5.27	19.38 ± 3.81	−4.633	< 0.001
Withdrawal/depression	5.67 ± 3.27	11.75 ± 2.60	−4.923	< 0.001
Social problems	3.67 ± 2.95	9.75 ± 4.52	−4.818	< 0.001
Thought problems	3.38 ± 3.56	11.75 ± 7.46	−4.894	< 0.001
Attention problems	5.10 ± 2.99	9.25 ± 3.95	−3.375	< 0.001
Rule-breaking behavior	1.64 ± 1.71	9.13 ± 10.99	−4.179	< 0.001
Aggressive behavior	5.95 ± 2.97	11.13 ± 6.53	−3.551	< 0.001
Internalizing problems	19.69 ± 10.10	38.38 ± 10.50	−4.733	< 0.001
Externalizing problems	7.59 ± 4.07	20.25 ± 6.791	−4.289	< 0.001
Total problems	43.28 ± 19.62	94.63 ± 36.07	−5.759	< 0.001
EDI-II				
Body dissatisfaction	6.65 ± 6.06	16.80 ± 11.58	−2.858	0.008
Interoceptive awareness	6.13 ± 7.33	15.80 ± 8.78	−2.586	0.015
Ascetism	4.74 ± 4.42	11.20 ± 7.22	−2.641	0.014
Social insecurity	4.61 ± 4.12	12.00 ± 8.42	−2.979	0.006

TCI = Temperament and character inventory; EDI-II = Eating disorder inventory – 2; ANT0 = AN without NSSI at T0; NANT0 = AN with NSSI at T0

The AN comorbid with NSSI at intake group scored higher in Anxiety/Depression (*p* < 0.001), Withdrawal/Depression (*p* < 0.001), Social Problems (*p* < 0.001), Thought Problems (*p* < 0.001), Attention Problems (*p* < 0.002), Rule-Breaking Behavior (*p* < 0.001), Aggressive Behavior (*p* < 0.001), Internalizing Problems (*p* < 0.001), Externalizing Problems (*p* < 0.001), and Total Problems (*p* < 0.001) at the YSR.

The AN comorbid with NSSI at intake group showed significantly higher mean scores in Body Dissatisfaction (*p* < 0.008), Interoceptive Awareness (*p* < 0.015), Asceticism (*p* < 0.014) and Social Insecurity (*p* < 0.006) at the EDI-II.

### AN comorbid with NSSI at intake vs AN reporting NSSI episodes between T0 and T1

No significant difference regarding the weekly frequency of NSSI and the total number of NSSI methods used was evidenced between the AN comorbid with NSSI at intake and the AN reporting NSSI episodes between T0 and T1 groups.

The AN comorbid with NSSI at intake group displayed a significantly higher BMI (*p* < 0.014) compared to the AN reporting NSSI episodes between T0 and T1 participants.

No significant differences between the AN comorbid with NSSI at intake and the AN reporting NSSI episodes between T0 and T1 groups were found among TCI and EDI-II. AN comorbid with NSSI at intake reported higher scores in Anxiety (*p* < 0.005), Attention Problems (*p* < 0.005), Internalizing Problems (*p* < 0.013), and Total Problems (*p* < 0.006) at the YSR.

### AN vs AN with NSSI

No significant difference was found regarding the subtype of AN, the age of onset of AN, the length of hospitalization, the mean BMI at the time of admission or the presence of Axis I disorders between the AN and the AN with NSSI groups.

The AN with NSSI group showed significantly lower Reward Dependence (RD) (*p* < 0.001), Determination (SD2) (*p* < 0.017) and Cooperativeness (C) (*p* < 0.013) at the TCI (Table 5).

**Table 5:** Student’s *t* test comparison of psychodiagnostics features between AN and AN with NSSI

TCI	AN (*n* = 27) mean ± sd	AN with NSSI (*n* = 19) mean ± sd	*t*	*p*
Reward dependence (RD)	15.00 ± 3.53	10.55 ± 3.38	3.567	0.001
Determination (SD2)	5.56 ± 1.59	3.90 ± 0.87	2.769	0.017
Cooperativeness (C)	33.78 ± 5.17	27.45 ± 9.69	2.624	0.013
YSR				
Activities	6.83 ± 4.01	9.85 ± 2.81	−2.551	0.014
Anxiety/depression	10.30 ± 5.36	15.36 ± 6.42	−2.585	0.017
Withdrawal/depression	5.36 ± 3.09	9.86 ± 3.90	−3.831	0.001
Social problems	3.48 ± 3.08	7.57 ± 4.42	−3.152	0.005
Thought problems	3.42 ± 3.68	8.07 ± 7.29	−2.914	0.006
Rule-breaking behavior	1.61 ± 1.64	6.00 ± 9.00	−2.737	0.009
Internalizing problems	19.36 ± 10.16	31.14 ± 13.31	−2.966	0.008
Externalizing problems	7.76 ± 3.88	14.43 ± 14.54	−2.469	0.017
Total problems	43.55 ± 20.28	72.00 ± 39.34	−3.280	0.002
EDI-II				
Body dissatisfaction	6.14 ± 5.66	17.00 ± 10.37	−3.454	0.002
Ineffectiveness	4.32 ± 4.88	10.67 ± 6.86	−2.591	0.016
Interpersonal insecurity	4.55 ± 3.80	10.33 ± 6.22	−2.875	0.008
Ascetism	4.64 ± 4.50	10.50 ± 6.69	−2.549	0.017
Social insecurity	4.14 ± 3.52	12.50 ± 7.64	−3.941	0.001

The AN with NSSI group showed significantly higher Activities (*p* < 0.014), Anxiety/Depression (*p* < 0.017), Withdrawal/Depression (*p* < 0.001), Social Problems (p < 0.005), Thought Problems (*p* < 0.006), Rule-Breaking Behavior (*p* < 0.009), Internalizing Problems (*p* < 0.008), Externalizing Problems (*p* < 0.017) and Total Problems (*p* < 0.002) at the YSR (Table 5).

The AN with NSSI group showed significantly higher Body Dissatisfaction (*p* < 0.002), Ineffectiveness (*p* < 0.016), Interpersonal Insecurity (*p* < 0.008), Asceticism (*p* < 0.017) and Social Insecurity (*p* < 0.001) at the EDI-II (Table 5).

### Follow-up

At follow-up, the remission rate of AN in the AN with NSSI group was significantly lower (*p* < 0.001) compared to the AN group (Table 3). No significant difference was found between the two groups at the TCI and YSR.

AN with NSSI group displayed significantly higher scores in Interpersonal Insecurity (*p* < 0.003) and Asceticism (*p* < 0.001) at the EDI-II.

The T1–T0 Delta of BMI, TCI, and EDI-II did not differ significantly between the two groups.

## Discussion

The present study aimed at a follow-up of a sample of adolescent inpatients with AN, with or without comorbid NSSI, to evidence the correlates of the NSSI attitudes with respect to clinical and psychopathological features and 5-year outcome.

The prevalence of NSSI at T0 in the sample was 25%, consistent with data reported in the literature [2]. At follow-up, it emerged that among those who had only an AN diagnosis at T0 21% engaged in NSSI in the period following hospitalization. The overall prevalence of NSSI in our sample (34%) was, therefore, in line with the literature which reports highly variable prevalence estimates depending on additional factors, such as eating disorder subtype and treatment setting [2, 3].

The average age of onset of AN is 14 years in both participants with AN alone and AN comorbid with NSSI at intake groups. This finding diverges from the literature, which suggests that the average age of onset of AN is earlier in patients with NSSI [4]. This may be due to the inpatient treatment setting which is selected based on greater severity of the eating disorder.

The high frequency of comorbidity between AN and other psychiatric disorders, particularly mood disorders and OCD, is well-known [12–14]. At the time of hospitalization, 34% of participants were diagnosed with a comorbid major depressive episode (76%) or an obsessive–compulsive disorder (24%); nevertheless, no significant difference was evidenced between the AN comorbid with NSSI at intake and participants with AN alone groups suggesting that the NSSI expression is rather independent from comorbid psychopathology.

AN comorbid with NSSI at intake group participants displayed greater psychopathological severity of the eating disorder in comparison with the AN alone participants, displaying higher body dissatisfaction, deficits of interoceptive awareness, greater asceticism and worse social insecurity. As hypothesized by existing literature, this was not associated with a worse state of malnutrition but with greater psychopathological suffering [1, 5].

Consistently with literature findings AN comorbid with NSSI at intake adolescents presented significantly lower cooperation and social feeling skills at the TCI [1]. Lower cooperation and social feeling align with the tendency of adolescents with NSSI toward relational disease and social isolation, which is both a consequence and a risk factor for the NSSI itself [15]. Our findings strengthen this evidence because of a lower dependence on social reward in the AN comorbid with NSSI at intake group. This is consistent with the literature, which indicates that low dependence on social reward in AN participants is associated with greater general and eating psychopathology and greater resistance to treatment [16].

AN comorbid with NSSI at intake reported significantly higher psychopathology at the YSR. High levels of anxiety, depression, somatic complaints, and aggression are part of the internalizing and externalizing problems typical of these patients [17]. Our findings also confirm the presence of greater relational and emotional issues in AN participants with NSSI, which manifest as significant difficulties in interpersonal relationships, expressed through feelings of inadequacy and anger toward others [18].

The AN comorbid with NSSI at intake and the AN reporting NSSI episodes between T0 and T1 groups displayed no significant differences in compensatory physical activity, BMI at admission and duration of hospitalization. Nevertheless, AN comorbid with NSSI at intake adolescents had significantly higher anxiety levels, greater social withdrawal and social problems, the most severe thought problems, greater attention problems, a tendency toward aggression, deeper internalizing problems, and worse general psychopathology problems while showing similar eating psychopathology characteristics to AN reporting NSSI episodes between T0 and T1. It is thus evident that AN comorbid with NSSI at intake adolescents expressed their NSSI in relationship with their more compromised overall functioning which was not strictly related to eating psychopathology [1]. We can hypothesize that, despite their less severe general functioning impairment, AN reporting NSSI episodes between T0 and T1 expressed their NSSI because of the modeling effect of the inpatient ward environment [19].

Among the 73 participants included in the study only 46 participated in the follow-up (63%). Participants and non-participants did not display any difference in the type of AN, presence of NSSI at T0, age of onset of the eating disorder, BMI, and duration of hospitalization. Therefore, the sample of participants has been considered representative of the entire target population. The overall remission rate of AN in the entire sample of participants (54.3%) was slightly higher than what is reported in the literature [16, 20, 21]. A recruitment bias could explain this observed difference: participants who did not consent to participate may still be ill and unwilling to discuss their illness, while those who have recovered may be more interested in sharing their story to help others.

In the AN with NSSI group only 21% of participants were in full remission compared to 78% of the AN group, while 37% of participants still met all the DSM-5 criteria [22] for AN compared to 7% of the AN group. This remission rate is less than one half of the mean of the whole sample, and less than that reported in the literature [16, 20, 21]. This evidence suggests that the comorbidity of NSSI with AN significantly worsens the AN prognosis and possibly influences the treatment effectiveness. As previously evidenced by follow-up studies [16] the presence of personality traits which impair relationality, as in the AN with NSSI, represents a strong indicator of treatment resistance in AN adolescents.

This evidence is reinforced by the fact that the majority of AN with NSSI participants (42%) are in partial remission, as they have a BMI above the diagnostic threshold (> 18.5) but still present dysfunctional eating thoughts and/or behaviors. This supports the evidence that in this group of adolescents affected with more severe psychopathological problems the mere increase of the weight may be reached without a remission of psychopathological troubles [5].

As concerns the evolution of the NSSI, at T1, these are still present in 31.5% of the participants. Among the adolescents who maintain their NSSI attitudes 50% display a stable AN which is the greatest rate of nonremission among our sample. Consistent with what has been reported in the literature [23] the average duration of NSSI ranges from 4 to 5 years, but considerable variability was observed (from a minimum of 6 months in a patient with O-NSSI to a maximum of 8 years). This further supports the fact that NSSI comorbidity, along with its specific psychopathology and personality features, represents a significant impairment in the healing process of AN [5, 16, 18].

### Strength and limits

This study has several limits. The initial sample size was sufficient to perform preliminary analyses; however, the low participation in the follow-up made it more difficult to assess the long-term progression as well as the evolution of the clinical and psychopathological characteristics. Furthermore, it is necessary to consider the possibility of selection bias, which may have influenced some of the results. In fact, the cohort comes entirely from a tertiary care center for child neuropsychiatric disease, where more complex and severe cases are likely to be referred. It is likely that most of the participants who agreed to participate have better personality traits and a more favorable current clinical condition, allowing them to share their illness history, while those who declined may still be ill, may have personality traits less inclined to cooperation, or may have distrust toward clinicians.

Due to the small sample size, it was not possible to differentiate the participants with NSSI into statistically sound subgroups that accounted for the variables related to the frequency and subtype of self-harm, and to the different subtypes of AN.

Limitations include a retrospective chart review, a lack of standardized NSSI measures, an absence of suicide attempt data, and the use of BMI instead of age-adjusted indices.

Despite these limitations, the study also presents several strengths. It is among the few studies examining the long-term outcome of adolescents with AN and comorbid NSSI after inpatient treatment. The use of standardized psychodiagnostic instruments (TCI, YSR, EDI-II) at both baseline and follow-up provides a comprehensive assessment of personality, behavioral, and psychopathological dimensions. The 5-year observation period allows for evaluation of illness trajectories and remission patterns.

### What is already known on this subject?

Anorexia nervosa (AN) and non-suicidal self-injury (NSSI) often co-occur in adolescents. The presence of NSSI in participants with AN has been associated with greater psychological distress, higher psychopathological severity, and longer treatment duration. Previous research indicates that AN adolescents with NSSI exhibit specific personality traits, as well as more severe emotional and relational difficulties. However, few longitudinal studies have examined how NSSI comorbidity influences the long-term clinical course and recovery of AN.

### What does this study add?

This 5-year follow-up study demonstrates that adolescents with AN and comorbid NSSI show a significantly worse long-term outcome compared to those with AN alone. Participants with AN and NSSI presented lower levels of Reward Dependence and Cooperativeness and higher levels of emotional and behavioral dysfunction. The remission rate was significantly lower in the AN + NSSI group (21%) than in the AN-only group (78%). These findings highlight the need for early identification of NSSI in AN participants and for tailored, integrative therapeutic approaches to address emotional dysregulation and self-harming behaviors.

## Conclusion

The comparison between the two groups showed that the difference in remission rates is statistically significant, with AN with NSSI adolescents displaying a less positive outcome compared to participants with AN only. This confirms what was previously observed in the literature: in AN with NSSI participants the lower levels of cooperation and social feeling, and dependence on social reward, associated with high emotional dysregulation, affect not only the initial psychopathological severity of the eating disorder but also its long-term clinical course.

These findings suggest that the presence of NSSI may represent a marker of greater emotional dysregulation and interpersonal difficulties, highlighting the importance of early identification and tailored interventions.

Since the general functioning of AN with NSSI was significantly more impaired compared to participants with only AN, these adolescents require treatment to be appropriately tailored, utilizing specific therapies such as DBT-A (Dialectical Behavioral Therapy for Adolescents) [24], CBT-E (Enhanced Cognitive Behavioral Therapy) [22], or MBT-A (Mentalization-Based Therapy for Adolescents) [25] that focus on behavioral and emotional aspects connected to the NSSI behaviors.

## Data Availability

Raw data of the study are available upon reasonable request to the corresponding author.
